# Waitlist mortality and post transplant survival amongst non-extracorporeal membrane oxygenation high-urgency heart transplant recipients before and after the 2018 policy change

**DOI:** 10.1016/j.xjon.2026.101936

**Published:** 2026-06-20

**Authors:** John P. White, Joseph Song, Samuel T. Kim, Hedwig Zappacosta, Peter D. Cho, Stephanie McKay, Akanksha Girish, Alexey Abramov, Malini Daniel, Donatello Telesca, Majid Husain, Abbas Ardehali

**Affiliations:** a David Geffen School of Medicine, UCLA, Los Angeles, Calif; b Division of Cardiovascular Surgery, Department of Surgery, University of Pennsylvania, Philadelphia, Pa; c Drexel University College of Medicine, Philadelphia, Pa; d Department of Surgery, Stamford Hospital, Stamford, Conn; e Division of Cardiac Surgery, Department of Surgery, UCLA, Los Angeles, Calif; f Department of Biostatistics, UCLA, Los Angeles, Calif; g Division of Pediatric Cardiology, Department of Pediatrics, UCLA Mattel Children's Hospital, Los Angeles, Calif

**Keywords:** heart transplantation, organ qualities, donor characteristics, statistical modeling

## Abstract

**Objective:**

The adult heart allocation policy changed from a 3- to 6-tiered system in 2018, more precisely stratifying high-urgency patients. Many studies examined the effects on patients classified as Status 1 or those with implantable devices, especially extracorporeal membrane oxygentation (ECMO), however, few have analyzed other high-urgency cohorts. This study's purpose is to compare waitlist and midterm outcomes of non-ECMO high-urgency patients before and after the policy change.

**Methods:**

All high-risk (Status 1A, 1, 2, 3) non-ECMO adult patients listed for isolated heart transplantation were identified using the United Network for Organ Sharing database and split based on the October 18, 2018, policy change date into era 1 (7/2014-7/2018) and era 2 (1/2019-1/2023). Categorical and continuous variables were assessed using χ^2^ and *t* tests, respectively. Waitlist mortality was analyzed using competing risk regression whereas Kaplan-Meier curves assessed posttransplant survival.

**Results:**

Candidates from era 2 demonstrated greater rates of comorbidities like pulmonary hypertension (65% vs 67%, *P* = .007) and greater need for support with inotropes (39% vs 47%, *P* < .001) and intra-aortic balloon pump (12% vs 42%, *P* < .001). Yet, candidates from era 2 had significantly decreased waitlist time (71 vs 19 days, *P* < .001) and waitlist mortality (19% vs 7%, *P* < .001) whereas Kaplan-Meier 3-year survival (86% vs 85%, *P* = .42) remained similar.

**Conclusions:**

Among non-ECMO high-urgency heart transplant recipients, candidates from era 2 demonstrated greater clinical acuity but improved waitlist mortality and equivalent midterm survival. These findings reassure that the 2018 policy change did not compromise outcomes amongst this high-urgency cohort, despite favoring patients bridged with ECMO.

**Figure:**
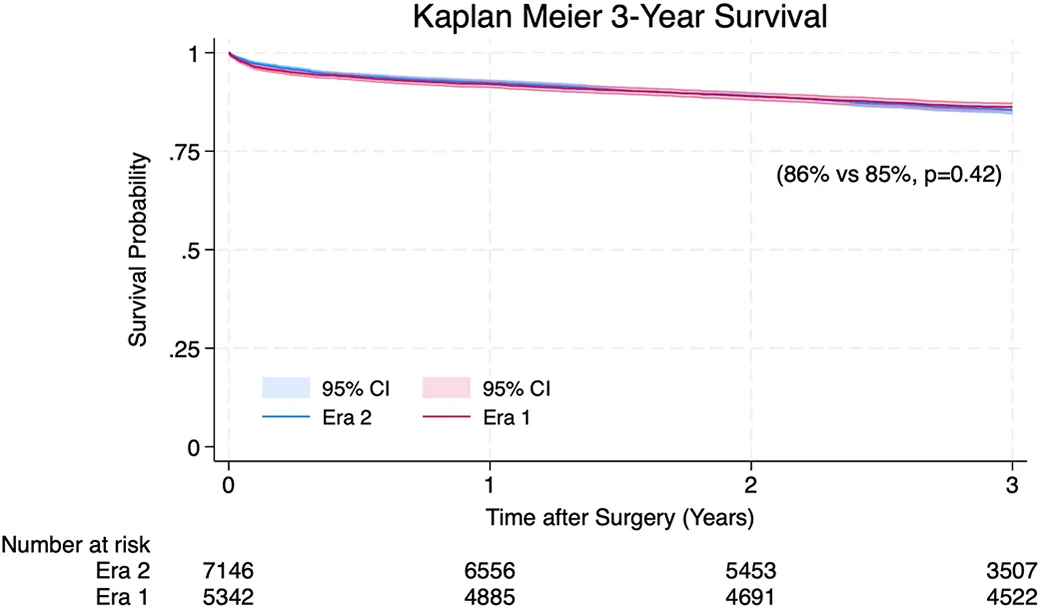
2018 heart allocation policy reduced waitlist mortality without compromising 3-y survival.

**Central Message**

The 2018 heart allocation policy reduced waitlist mortality and duration for non-ECMO high-urgency patients without compromising 3-year posttransplant survival.

**Perspective**

Despite prioritization of ECMO-supported patients, non-ECMO high-urgency candidates benefited from improved waitlist outcomes with preserved posttransplant survival. These findings suggest the revised allocation system enhances efficiency without disadvantaging competing high-risk populations, supporting its continued use and future refinement.

On October 18, 2018, the United Network for Organ Sharing (UNOS) implemented a change in the heart allocation policy, moving from a 3-tiered (1A, 1B, 2) to a 6-tiered (1, 2, 3, 4, 5, 6) system with the goal of more precisely stratifying patients with the greatest clinical urgency awaiting heart transplant.^1^^,^^2^ In large part, the policy separated tier 1A into tiers 1, 2, and 3 to better address the needs of the patients with the greatest acuity. The new policy achieved this by prioritizing patients with hospital-based temporary mechanical support (ie, extracorporeal membrane oxygenation [ECMO], intra-aortic balloon pump [IABP], and percutaneous devices) or inotrope dependence while deprioritizing stable, dischargeable patients with durable left ventricular assist devices (LVADs), resulting in a shift in the profile of patients being transplanted, which has been a large focus of postpolicy change research.^3^, ^4^, ^5^

Before the allocation policy change, several UNOS registry studies suggested that candidates on ECMO had lower early and midterm survival compared with all heart transplant recipients.^6^^,^^7^ However, subsequent analysis of UNOS registry data in the postpolicy era demonstrated that survival of ECMO-bridged candidates appeared to be comparable with that of all other heart transplant recipients.^5^^,^^8^^,^^9^ In addition, several studies analyzed the broader impact of the new system on waitlist mortality, transplant rate, and survival. Earlier studies suggested decreased posttransplant survival^2^^,^^10^^,^^11^; however, more recent studies alluded that the new allocation system leads to better stratification of medically urgent candidates with improved transplant rate and unchanged posttransplant survival broadly and amongst the ECMO cohort.^5^^,^^12^, ^13^, ^14^

Because of the prioritization of candidates supported by ECMO, this group now comprises a large proportion of Status 1 recipients, and much of the existing literature has focused on this narrow cohort. In contrast, there remains a relative paucity of data examining outcomes among other high-priority, non-ECMO candidates across status levels. Previous studies evaluating shifts in mechanical support use have often been limited to single-device analyses or smaller subsets, reducing their generalizability.^15^ Therefore, a larger contemporary cohort study is warranted to ensure that the non-ECMO, high-urgency recipients are served in a timely fashion with acceptable waitlist mortality and outcomes.

The objective of this study was to evaluate non-ECMO high-priority candidates (pre-2018 Status 1A; post-2018 Statuses 1-3) before and after the 2018 allocation change, focusing on candidate characteristics, waitlist outcomes, and 3-year posttransplant survival.

## Methods

### Study Population

A retrospective registry analysis of the UNOS database was performed for all adults (≥18 years old) undergoing heart transplantation from July 2014 to January 2023. Multiorgan transplants and all patients on ECMO at any point during the waitlist were excluded. All patients listed from July 2014 to July 2018 were considered era 1, whereas those listed from January 2019 to January 2023 were considered era 2. July 2018 to January 2019 was excluded from analysis to provide a 6-month washout period surrounding the October 18, 2018 policy change date to account for any crossover effect (Figure 1). Era 1 was composed of non-ECMO pre-2018 Status 1A patients, whereas era 2 contained non-ECMO patients with post-2018 Statuses 1, 2, and 3. Patients within the study cohort are referred to as non-ECMO high-urgency candidates. More patients were observed at transplant than listing because more patients are upgraded to Status 1 to 3 after listing, adding them to the cohort, than those placed on ECMO after listing, which removes them from cohort. Status was defined at the time of listing for all waitlist analyses, whereas status at the time of transplantation was used for analysis of posttransplant outcomes. The primary study outcome was 3-year posttransplant survival. Secondary end points included waitlist mortality and predictors of 3-year posttransplant mortality in patients in era 2. This study was deemed exempt from review by the institutional review board at the University of California, Los Angeles, and there was no requirement for informed consent.

**Figure 1:**
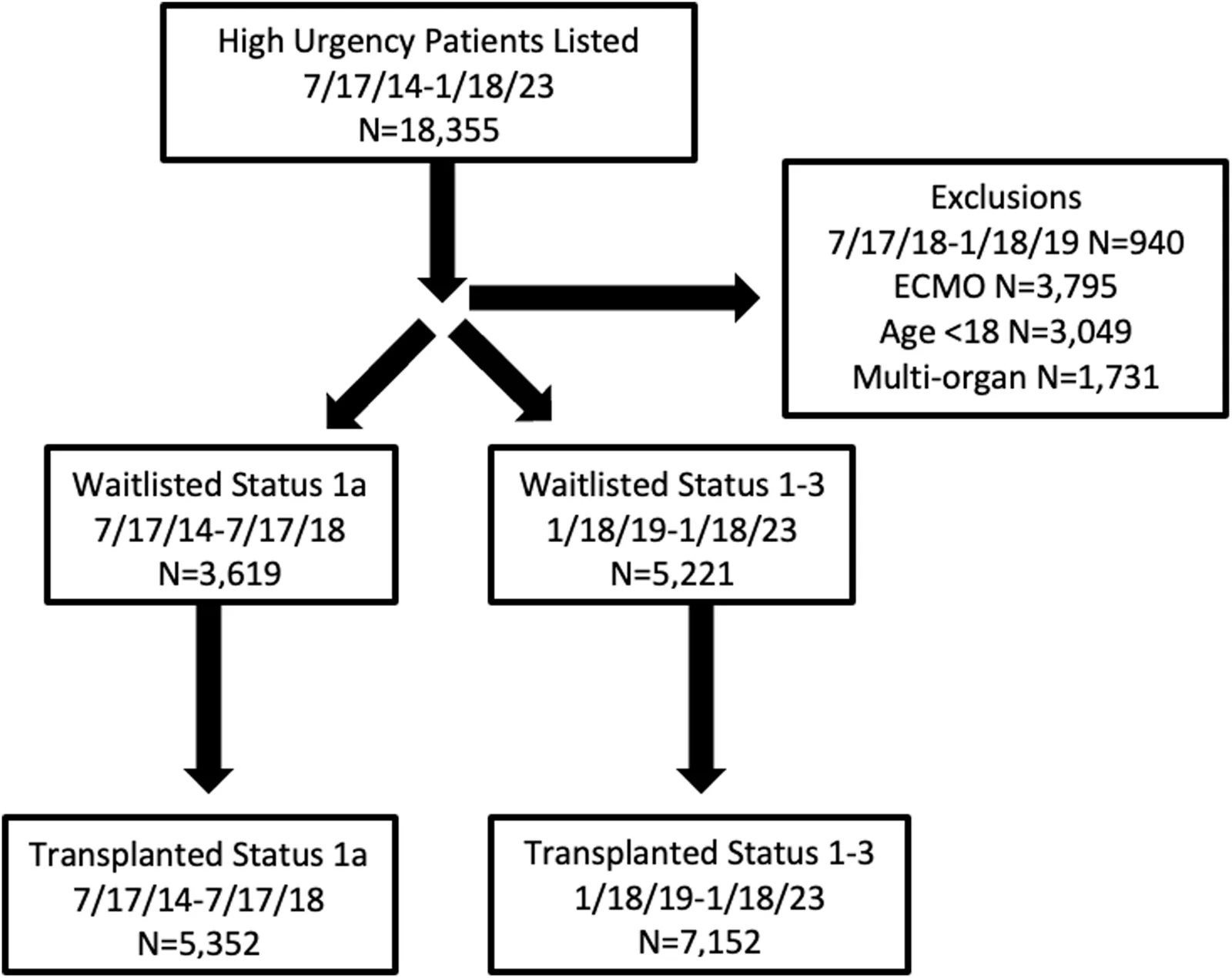
Consolidated Standards of Reporting Trials diagram of the study population. *ECMO*, Extracorporeal membrane oxygenation.

### Candidate Characteristics

Several factors were compared between patients in era 1 and era 2 who were waitlisted for heart transplantation: age, sex, body mass index (BMI), minority status, comorbidities (cerebrovascular disease, diabetes, pulmonary hypertension), previous cardiac surgery, use of mechanical circulatory support at transplantation (IABP, temporary LVAD), inotrope or ventilator use at transplantation, serum total bilirubin, cardiac output, and waitlist death or deterioration. The factors analyzed for candidates in eras 1 and 2 included all previously mentioned waitlist factors plus serum creatinine, blood urea nitrogen, size mismatch defined by predicted heart mass,^15^ ischemic time, donor to recipient distance, length of stay to discharge, and mortality. Donor factors compared between patients in era 1 and era 2 included age, sex, donor race, BMI category, diabetes, hypertension, cigarette use, alcohol use, cause of death, bilirubin, creatinine, and left ventricular ejection fraction.

### Statistical Analyses

For categorical variables, groups were compared using frequencies with percentages and the χ^2^ test. Continuous variables are displayed as the mean with interquartile range and compared using the *t* test. All *P* values were derived from 2-sided tests, with a threshold of *P* < .05 indicating statistical significance. All variables are presented with the era 1 value first, era 2 value second, followed by the *P* value (ie, era 1%, era 2%, *P* < .001).

Competing risk regression was used to compare waitlist mortality. Multivariable logistic regression was used to determine factors associated with waitlist death/deterioration and 3-year posttransplant survival in separate models. The 3-year model was contingent on survival at 30 days. Factors associated with mortality are displayed as adjusted odds ratios with 95% CIs for all models. A two-tailed *P*-value <.05 was considered significant for all analyses.

Kaplan-Meier survival estimates were used to visualize survival, with the log-rank test used to assess for significant differences. Sub-group analyses was performed to assess differences in survival between non-ECMO status 1 and 2 patients within era 2. All analyses were performed using Stata 19.5 (StataCorp).

## Results

### Characteristics of Era 1 and 2 Candidates at Listing

Characteristics of era 1 and era 2 candidates at listing for heart transplantation are displayed in Table 1. Patients in era 1 listed for heart transplantation were more likely to have increased BMI (27.0 vs 26.8 kg/m^2^, *P* < .001), previous cardiac surgery (38% vs 32%, *P* < .001), temporary LVAD support at listing (42% vs 34%, *P* < .001), ventilator dependence at listing (7% vs 2%, *P* < .001), and greater rates of waitlist death or deterioration (19% vs 7%, *P* < .001). In contrast, patients in era 2 undergoing heart transplantation were more likely to have diabetes (27% vs 29%, *P* = .007), pulmonary hypertension (69% vs 72%, *P* = .006), and be on an IABP at listing (15% vs 33%, *P* < .001).

**Table 1:** Characteristics of non-ECMO high-urgency heart transplant candidates at listing by era

Variable	Era 1, n = 3619	Era 2, n = 5221	*P* value
Age, y	56 (44-63)	56 (45-63)	.56
Female	977 (27)	1330 (25)	.11
BMI, kg/m^2^	27.0 (23.6-31.1)	26.8 (23.2-30.8)	<.001
Minority status	1478 (41)	2307 (44)	.002
Cerebrovascular disease	264 (7)	422 (8)	.17
Diabetes	969 (27)	1536 (29)	.007
Pulmonary hypertension∗	2510 (69)	3763 (72)	.006
Previous cardiac surgery	1365 (38)	1682 (32)	<.001
IABP at listing	525 (15)	1739 (33)	<.001
Temporary LVAD at listing	1514 (42)	1751 (34)	<.001
Inotrope use at listing	1606 (44)	2323 (44)	.91
Ventilator use at listing	238 (7)	84 (2)	<.001
Serum total bilirubin at listing, mg/dL	0.7 (0.5-1.2)	0.7 (0.5-1.1)	.08
Cardiac output at listing, L/min	4 (3.2-5)	4 (3.2-5)	.11
1-y waitlist death or deterioration	686 (19)	385 (7)	<.001

Values are expressed as frequency (proportion of population) or median (interquartile range). *P* values for *t* test or χ^2^ tests for continuous and categorical variables, respectively. *ECMO*, Extracorporeal membrane oxygenation; *BMI*, body mass index; *IABP*, intra-aortic balloon pump; *LVAD*, left ventricular assist device; *PA*, pulmonary artery.

∗ Pulmonary hypertension defined as mean PA pressure ≥25 mm Hg.

### Waitlist Regression Analysis

Competing risk regression displays decreased waitlist death or deterioration in era 2 when compared with era 1 (19% vs 7%, *P* < .001) (Figure 2, *A*). Factors associated with death or deterioration requiring delisting among heart transplant candidates among patients from era 2 are shown in Table 2. Among candidates for heart transplant from era 2, only female candidates (0.71, 0.55-0.93, *P* = .01) and IABP at listing (0.73, 0.58-0.93, *P* = .009) were associated with decreased waitlist death or deterioration. The factors associated with increased risk for waitlist mortality were increased age (1.02 per year, 1.01-1.03, *P* < .001), increased BMI (1.02 per kg/m^2^, 1.00-1.04, *P* = .03), pulmonary hypertension (1.47, 1.14-1.89, *P* = .003), temporary LVAD at listing (1.74, 1.19-2.25, *P* = .005), ventilator use at listing (3.81, 2.26-6.42, *P* < .001), and increased cardiac output (1.08 per L/min, 1.01-1.16, *P* = .03).

**Figure 2:**
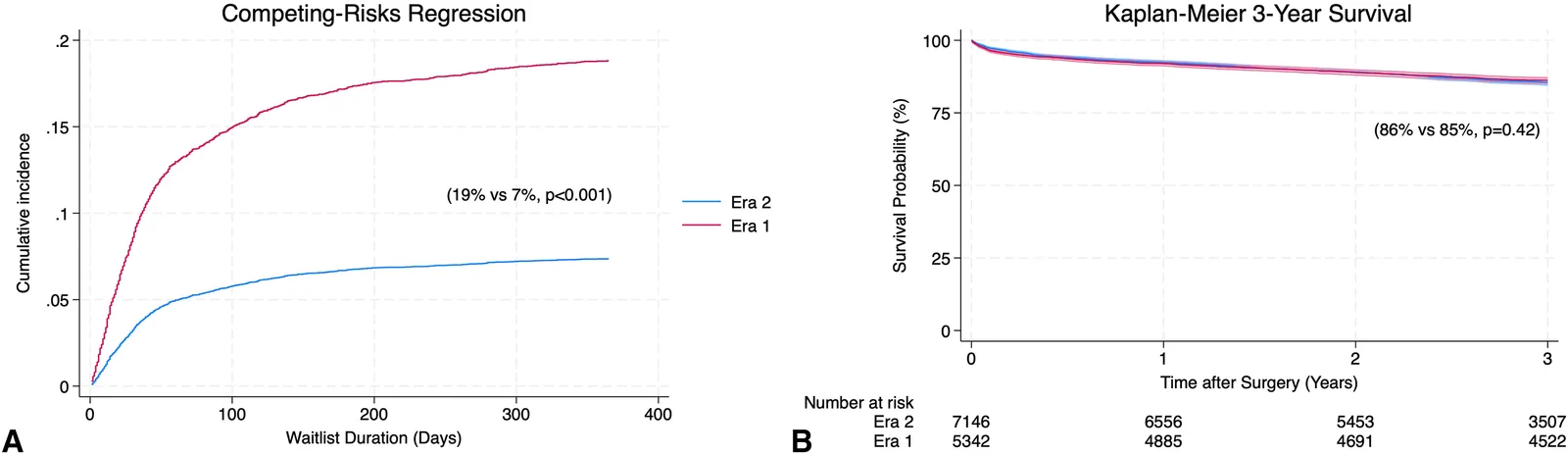
A, Competing risks regression of waitlist death or deterioration amongst non-ECMO high-urgency patients from era 1 and era 2. B, Kaplan-Meier 3-year posttransplant survival amongst non-ECMO high-urgency patients from era 1 and era 2. *ECMO*, Extracorporeal membrane oxygenation.

**Table 2:** Risk prediction model for waitlist death or deterioration in era 2 candidates listed for heart transplantation

Variable	Adjusted odds ratio	95% CI	*P* value
Age, per 1 y	1.02	1.01-1.03	<.001
Female	0.71	0.55-0.93	.01
Body mass index, per kg/m^2^	1.02	1.00-1.04	.03
Alcohol use	1.09	0.89-1.35	.40
Diabetes	1.20	0.96-1.49	.11
Cerebrovascular disease	1.11	0.77-1.60	.58
Pulmonary hypertension	1.47	1.14-1.89	.003
Previous cardiac surgery	1.24	1.00-1.53	.06
IABP at listing	0.73	0.58-0.93	.009
Temporary LVAD at listing	1.74	1.19-2.25	.005
Ventilator use at listing	3.81	2.26-6.42	<.001
Inotropes at listing	1.05	0.86-1.30	.62
Cardiac output, per L/min	1.08	1.01-1.16	.03

Pulmonary hypertension defined as mean PA pressure ≥ 25 mm Hg. *IABP*, Intra-aortic balloon pump; *LVAD*, left ventricular assist device; *PA*, pulmonary artery.

### Characteristics of Donors From Eras 1 and 2

Characteristics of era 1 and era 2 heart donors are displayed in Table 3. Donors from era 1 were more likely to be female (28% vs 24%, *P* < .001) and have died of a head trauma (47% vs 41%, *P* < .001) when compared with patients from era 2. Donors from era 2, in contrast, were more likely to be older (31 vs 30 years, *P* = .005) and have died from anoxia (45% vs 35%, *P* < .001). Both groups of donors had a similar history of hypertension (16% vs 14%, *P* = .13), diabetes (4% vs 3%, *P* = .14), and left ventricular ejection fraction (60% vs 60%, *P* = .73). Donation after circulatory death was significantly more common among donors in era 2 (0% vs 4%, *P* < .001).

**Table 3:** Characteristics of non-ECMO high-urgency heart donors by era

Variable	Era 1, n = 5352	Era 2, n = 7152	*P* value
Donor characteristics			
Age, y	30 (23-39)	31 (24-39)	.005
Female	1489 (28)	1684 (24)	<.001
Donor race			.08
White	3377 (63)	4388 (61)	
Black	901 (17)	1201 (17)	
Hispanic	921 (17)	1344 (19)	
Asian	89 (2)	110 (2)	
Other	64 (1)	109 (2)	
BMI category			.09
No obesity	3721 (70)	4843 (68)	
Obesity	1540 (29)	2189 (31)	
Underweight	91 (2)	120 (2)	
Diabetes	193 (4)	224 (3)	.14
Hypertension	848 (16)	1010 (14)	.13
Cigarette use	545 (10)	789 (11)	.13
Alcohol use	911 (17)	1311 (18)	.06
Cause of death			<.001
Anoxia	1869 (35)	3233 (45)	
Cerebrovascular/stroke	847 (16)	818 (11)	
Head trauma	2494 (47)	2933 (41)	
CNS tumor	25 (1)	21 (0.3)	
Other	117 (2)	147 (2)	
Bilirubin, mg/dL	0.7 (0.5-1.1)	0.7 (0.5-1.1)	.10
Creatinine, mg/dL	1.0 (0.8-1.5)	1.0 (0.8-1.6)	.08
LVEF, %	60 (56-65)	60 (56-65)	.73
Donation after circulatory death	0 (0)	278 (4)	<.001

Values are expressed as frequency (proportion of population) or median (interquartile range). *P* values for *t* test or χ^2^ tests for continuous and categorical variables, respectively. *ECMO*, Extracorporeal membrane oxygenation; *BMI*, body mass index; *CNS*, central nervous system; *LVEF*, left ventricular ejection fraction.

### Characteristics of Candidates From Eras 1 and 2 at Transplant

Characteristics of candidates from era 1 and era 2 at heart transplant are displayed in Table 4. All statistically significant variables from listing (Table 1) remained significant, except BMI (27.3 vs 27.3 kg/m^2^, *P* = .08), diabetes (28% vs 29%, *P* = .42), and ventilator use (1% vs 1%, *P* = .26). Additionally, candidates from era 1 were more likely to have increased blood urea nitrogen (23 vs 21 mg/dL, *P* < .001), gender mismatch (13% vs 9%, *P* < .001), ischemic time (3.5 vs 3.4 hours, *P* < .001), and donor-to-recipient distance (262 vs 192 miles, *P* < .001). Importantly, both the number of days on the waitlist and duration of posttransplant hospital stay were longer in candidates from era 1 (71 vs 19 days, *P* < .001; 17 vs 16 days, *P* < .001, respectively). Candidates from era 2 were more likely to have cerebrovascular disease (7% vs 8%, *P* = .002), greater inotropic use at transplantation (39% vs 47%, *P* < .001), serum creatinine (1.1 vs 1.2 mg/dL, *P* = .04), and cardiac output (4.2 vs 4.3 L/min, *P* < .001).

**Table 4:** Characteristics of non-ECMO high-urgency heart transplant candidates at transplant by era

Variable	Era 1 n = 5352	Era 2 n = 7152	*P* value
Recipient characteristics			
Age, y	56 (46-63)	56 (46-63)	.92
Female	1290 (24)	1754 (25)	.59
BMI, kg/m^2^	27.3 (23.9-31.3)	27.3 (23.9-31.1)	.08
Cerebrovascular disease	356 (7)	579 (8)	.002
Diabetes	1508 (28)	2062 (29)	.42
Pulmonary hypertension∗	3471 (65)	4803 (67)	.007
Previous cardiac surgery	2181 (41)	2427 (34)	<.001
IABP at transplant	638 (12)	2970 (42)	<.001
Temporary LVAD at transplant	2567 (48)	2338 (33)	<.001
Inotrope use at transplant	2099 (39)	3388 (47)	<.001
Ventilator use at transplant	42 (1)	70 (1)	.26
Serum creatinine, mg/dL	1.1 (0.9-1.4)	1.2 (0.9-1.4)	.04
Serum total bilirubin, mg/dL	0.7 (0.5-1.1)	0.7 (0.5-1.1)	.10
Blood urea nitrogen, mg/dL	23 (16-36)	21 (14-33)	<.001
Cardiac output, L/min	4.2 (3.3-5.2)	4.3 (3.4-5.3)	<.001
Waitlist duration, d	71 (23-197)	19 (7-63)	<.001
Donor-recipient matching			
Size mismatch†	1184 (22)	1521 (21)	.25
Gender mismatch	711 (13)	668 (9)	<.001
Ischemic time, h	3.5 (3.0-4.1)	3.4 (2.7-3.9)	<.001
Donor to recipient distance, miles	262 (119-421)	192 (44-372)	<.001
Length to discharge, d	17 (12-25)	16 (12-24)	<.001
Mortality			
30-d	176 (3)	183 (3)	.20
1-y	437 (8)	555 (8)	.41
3-y	742 (14)	961 (13)	.49

Values are expressed as frequency (proportion of population) or median (interquartile range). *P* values for *t* test or χ^2^ tests for continuous and categorical variables, respectively. *ECMO*, Extracorporeal membrane oxygenation; *BMI*, body mass index; *IABP*, intra-aortic balloon pump; *LVAD*, left ventricular assist device; *PA*, pulmonary artery.

∗ Pulmonary hypertension defined as mean PA pressure ≥25 mm Hg.

† Size mismatch defined as donor predicted heart mass ≤80% recipient predicted heart mass.

### Survival Characteristics

Kaplan-Meier demonstrated similar 3-year posttransplant survival among candidates from eras 1 and 2 (86% vs 85%, *P* = .42) (Figure 2, *B*). Predictors of 3-year mortality conditional on 30-day survival among Era 2 patients are shown in Table 5. After adjustment, age (1.01 per year, 1.00-1.01, *P* = .004), BMI (1.03 per kg/m^2^, 1.01-1.04, *P* < .001), diabetes (1.19, 1.04-1.36, *P* = .01), previous cardiac surgery (1.46, 1.28-1.66, *P* < .001), temporary LVAD at transplantation (1.24, 1.09-1.41, *P* = .001), ventilator use at transplantation (1.95, 1.21-3.15, *P* = .006), increased serum creatinine (1.14 per mg/dL, 1.09-1.19, *P* < .001), total bilirubin (1.05 per mg/dL, 1.03-1.07, *P* < .001), ischemic time (1.06 per hour, 1.00-1.12, *P* = .04), and waitlist duration (1.02 per day, 1.01-1.03, *P* < .001) were associated with increased odds of mortality at 3 years after transplantation. Notably, IABP use at transplant was not associated with increased mortality (0.88, 0.78-1.01, *P* = .06; 0.83, 0.62-1.11, *P* = .20, respectively). History of cerebrovascular disease and pulmonary hypertension were also not associated with increased mortality (0.97, 0.77-1.23, *P* = .83; 0.98, 0.85-1.12, *P* = .72, respectively).

**Table 5:** Risk prediction model for 3-year mortality in era 2 heart transplant recipients

Variable	Adjusted odds ratio	95% CI	*P* value
Age, per y	1.01	1.00-1.01	.004
Female	1.13	0.98-1.31	.09
BMI, per kg/m^2^	1.03	1.01-1.04	<.001
Cerebrovascular disease	0.97	0.77-1.23	.83
Diabetes	1.19	1.04-1.36	.01
Pulmonary hypertension∗	0.98	0.85-1.12	.72
Previous cardiac surgery	1.46	1.28-1.66	<.001
IABP at transplant	0.88	0.78-1.01	.06
Temporary LVAD at transplant	1.24	1.09-1.41	.001
Inotrope use at transplant	0.93	0.82-1.06	.27
Ventilator use at transplant	1.95	1.21-3.15	.006
Serum creatinine, per mg/dL	1.14	1.09-1.19	<.001
Serum total bilirubin, per mg/dL	1.05	1.03-1.07	<.001
Cardiac output, per L/min	1.02	0.97-1.06	.47
Ischemic time, per hour	1.06	1.00-1.12	.04
Distance, per mile	1.00	1.00-1.00	.33
Size mismatch	0.99	0.85-1.16	.91
Waitlist duration, per d	1.02	1.01-1.03	<.001

*BMI*, Body mass index; *IABP*, intra-aortic balloon pump; *LVAD*, left ventricular assist device; *PA*, pulmonary artery.

∗ Pulmonary hypertension defined as mean PA pressure ≥25 mm Hg.

## Discussion

This study demonstrates that 3-year survival remains similar amongst non-ECMO, high-urgency candidates before and after the 2018 policy change whereas waitlist death or deterioration decreased in era 2. Collectively, this study provides valuable insight into the midterm effects of the 2018 policy change and provides reassuring evidence that the 2018 policy change did not negatively impact this cohort, despite giving greater prioritization to recipients of ECMO.

As expected from a change in allocation policy, our analysis reveals significant shifts in baseline characteristics of candidates for heart transplant between era 1 and era 2, namely in mechanical support use and markers of increased preoperative acuity. These findings reflect a fundamental change in the population being listed and transplanted after the policy implementation. The most obvious explanation is that the new policy preferentially allocated organs to greater-risk patients, in line with the original intent of prioritizing medical urgency. Another possible reason is that in the interim, new technologies were implemented more broadly, allowing for improved hemodynamic stabilization of critically ill patients and enabling them to remain transplant eligible. As a result, patients who previously may have deteriorated or been deemed unsuitable for transplant may now be successfully bridged to transplantation. Another possible explanation for the greater acuity of patients listed in era 2 is that transplant centers may have been more willing to list sicker patients, given greater confidence in prioritization and a greater likelihood of timely transplantation, thereby shifting the overall acuity profile of the waitlist.

The 2018 allocation policy was designed to prioritize the sickest patients, namely those on ECMO. One might therefore expect other high-urgency recipients who formerly shared the same status as patients bridged to ECMO to experience increased waitlist duration, greater mortality, or worse posttransplant survival; however, our findings demonstrate the opposite. Notably, non-ECMO high-urgency candidates in era 2 experienced lower waitlist mortality despite greater baseline illness severity. There are several possible explanations for this observed decrease. First, the new policy may represent a more precise allocation system that better aligns donor organ distribution with true medical urgency. By expediting transplantation for the sickest patients while allowing more stable candidates to wait safely, the policy may reduce the risk of waitlist death or deterioration across the cohort. Second, an increase in donor heart availability, as evidenced by increased era 2 transplants, may account for the difference. This increase coincides with expanded use of donation after circulatory death hearts, which has meaningfully altered the donor landscape and reduced competition among high-urgency candidates. Third, continued improvements in donor heart preservation and transport technologies may have increased transplant centers’ willingness to accept marginal or geographically distant organs, further expanding the effective donor pool. Lastly, advances in pretransplant medical management and bridge-to-transplant strategies likely contributed to improved stabilization of critically ill patients while awaiting transplantation.

Although candidates from era 2 had greater clinical acuity at both listing and transplant, 3-year survival remained comparable with that of candidates from era 1, suggesting that advances across multiple facets may be offsetting the increased baseline risk. First, substantial progress has been made in donor heart preservation strategies, including improved ischemic tolerance, enhanced organ transport logistics, and the increasing adoption of ex vivo perfusion technologies, all of which may contribute to improved early graft function and long-term durability. These advances likely allow transplant centers to safely use donor hearts that previously may have been declined, without compromising recipient outcomes. Second, continuous refinements in perioperative management, intensive care protocols, and posttransplant graft surveillance have likely improved early detection and management of complications, which may be particularly beneficial for more vulnerable, high-urgency recipients. Third, patients awaiting transplant are increasingly benefiting from optimized guideline-directed medical therapy and more sophisticated mechanical circulatory support strategies that preserve end-organ function and improve physiologic reserve before transplantation, potentially narrowing the survival gap between high- and lower-risk recipients. However, the deprioritizing of patients supported by LVADs is the subject of a current proposal before the Organ Procurement & Transplantation Network board to escalate status based on LVAD duration. Several studies have analyzed the impact of the 2018 policy change on LVAD waitlist mortality and outcomes, but it is an evolving topic.

This study has several key limitations. The use of a large national registry carries inherent risks, including missing data, reporting variability, and limited granularity regarding clinical decision-making. In addition, the UNOS records patient status only at listing and transplant, which prevents a more nuanced understanding of interim status changes. As a result, our study may not fully capture patients who were temporarily downgraded before death or those who transitioned between support modalities before transplantation. Our study found a seemingly paradoxical decrease in distance from donor to recipient, despite the policy change increasing the acceptable distance allowed for transplantation. This study period also encompasses the COVID-19 pandemic, which disrupted transplantation practices nationwide; however, national heart transplant volumes have largely recovered. Also, our study provides a descriptive analysis and does not propensity match or adjust for differences in baseline patient characteristics which may confound the final analysis. Finally, although data from the 6 months surrounding the policy change were censored to minimize crossover effects, this duration may be insufficient to fully account for anticipatory changes in physician behavior or institutional practice patterns.

In conclusion, among non-ECMO high-urgency candidates for heart transplant, patients listed after the 2018 allocation policy change demonstrated greater baseline clinical acuity yet experienced improved waitlist outcomes and equivalent midterm survival. These findings suggest that the 2018 policy change did not adversely impact outcomes in this high-urgency cohort, despite increased prioritization of recipients of ECMO.

## Conflict of Interest Statement

The authors reported no conflicts of interest.

The *Journal* policy requires editors and reviewers to disclose conflicts of interest and to decline handling or reviewing manuscripts for which they may have a conflict of interest. The editors and reviewers of this article have no conflicts of interest.
